# A Simple Physical Examination Predicts Cognitive Decline in Very Mild Dementia

**DOI:** 10.3390/jpm14111086

**Published:** 2024-11-01

**Authors:** Li-Han Lin, Karen Y. C. Chuang, Chung-Yao Hsu, Nai-Ching Chen, Jyun-Bin Huang, Hsiu-Yung Pan, Yao-Chung Chuang

**Affiliations:** 1 Department of Radiology, Kaohsiung Chang Gung Memorial Hospital, Chang Gung University College of Medicine, Kaohsiung 83301, Taiwan; 2 Department of Sports Medicine, College of Medicine, Kaohsiung Medical University, Kaohsiung 80708, Taiwan; 3 Department of Neurology, School of Medicine, College of Medicine, Kaohsiung Medical University Hospital, Kaohsiung Medical University, Kaohsiung 80708, Taiwan; 4 Department of Neurology, Kaohsiung Chang Gung Memorial Hospital, Kaohsiung 83301, Taiwan; 5 Department of Emergency Medicine, Kaohsiung Chang Gung Memorial Hospital, Kaohsiung 83301, Taiwan; 6 Division of Neurology, Department of Internal Medicine, Pao Chien Hospital, Pingtung City 90064, Taiwan

**Keywords:** aging, cognitive decline, dementia, mild cognitive impairment, physical fitness

## Abstract

Background: Different exercises have different effects upon physical fitness and cognitive domains. In this context, physical fitness behaviors have been identified as a contributing factor to cognitive decline in patients with very mild dementia. The present study aimed to further determine baseline senior fitness behaviors in patients with very mild dementia and possible factors related to rapid cognitive decline. Methods: This prospective cohort study was performed in a medical center in Taiwan, involving 132 patients with very mild dementia who were followed-up over 1 year. Assessments included the Senior Fitness Test (physical function), Mini-Mental State Examination (MMSE), and Clinical Dementia Rating (CDR) Scale. Patients with a decline in MMSE of at least 2 points within 1 year were defined as having rapid cognitive decline. Results: Age, sex, years of education, and baseline MMSE did not differ significantly between the groups (*p* > 0.05). At 1 year of follow-up, dietary habits and comorbidities did not differ between the rapid decline and not-rapid decline groups. At 1 year, performance on the right back scratch test was significantly better in the not-rapid decline group compared with the rapid decline group (−14 [−39–37.5] cm vs. −17 [−57–7] cm; *p* = 0.038). In a multiple regression analysis, the only factor that was significantly associated with rapid cognitive decline was the right back scratch test (*p* = 0.022). Conclusions: Despite the similarity in the status of dementia, the right hand back scratch test appears to serve an important function in detecting cognitive decline in patients with very mild dementia.

## 1. Introduction

According to the World Health Organization, the incidence of dementia grows exponentially with advancing age, and 9.9 million new cases of dementia are diagnosed worldwide every year [1]. During the process of normal aging, an incipient state termed mild cognitive impairment (MCI) or very mild dementia develops before the diagnosis of mild dementia [2]. Once a diagnosis of MCI is made, it is important for the clinician to determine whether the case is associated with the causes of cognitive impairment to avoid the high annual conversion rates of MCI developing into dementia, which range from 5% to 20% depending on the studied population [3]. Currently, no pharmacologic treatment has been proven to slow or cure the progression of MCI into dementia.

The diagnosis of very mild or mild dementia is based mainly on the patient’s medical history and cognitive examination, with supporting clinical examinations including imaging and laboratory tests. The main distinctions between these two forms of dementia are that mild dementia invariably involves more than one cognitive domain and the patient experiences substantial interference with daily life activities [3]. The prognosis for very mild or mild dementia is an important motivation for diagnosis because both conditions are associated with a heightened risk for further cognitive decline [4]. Thus, identifying or preventing factors associated with cognitive decline in patients with very mild dementia is a very important issue.

The beneficial effects of exercise in the prevention of cognitive decline have been investigated. For instance, 6 months of twice-weekly resistance training in older adult women with subjective memory complaints significantly improved Stroop Test (executive function) performance compared with balance and tone training [5], while in another study, 6 months of high-intensity aerobic exercise enhanced performance in executive function testing among older women at high risk of cognitive decline, whereas stretching had no such effects [6]. Accordingly, the evidence indicates that different exercises have different effects upon physical fitness and cognitive domains. The physical fitness behavior may be used as a factor for cognitive decline in patients with very mild dementia. Therefore, the present study aimed to further determine the exercise and exercise-related senior fitness behaviors in patients with very mild dementia, as well as to identify the potential factors associated with rapid cognitive decline.

## 2. Materials and Methods

### 2.1. Study Design and Participants

This prospective cohort study was conducted in accordance with the Declaration of Helsinki and was approved by the Institutional Review Board of Kaohsiung Chang Gung Memorial Hospital (Kaohsiung City, Taiwan) (IRB Approval No. 201900157B0). All study participants were treated in the multidisciplinary dementia outpatient clinic in Kaohsiung Chang Gung Memorial Hospital between February 2019 and February 2020.

The clinical diagnosis of each patient was interpreted by consensus of a panel consisting of neurologists, psychiatrists, neuropsychologists, and neuroradiologists working in the hospital’s dementia integrated outpatient clinic. Exclusion criteria included the diagnosis of cerebrovascular diseases, the inability to complete senior fitness testing, and requiring assistance to walk. A total of 132 participants (77 males, 55 females) diagnosed with very mild dementia (Clinical Dementia Rating [CDR] 0.5) and who had completed senior fitness examinations were enrolled in this study.

Clinicodemographic data including age, sex, educational level, and comorbidities (histories of operations in the knee, hip, or spine, and systemic diseases including diabetes, hypertension, hyperlipidemia, and gout), dietary habits, and vegetarian status were recorded for each study participant after enrollment. Dietary habits were defined by the participants’ responses to our questionnaire administered by a case manager, with the following question on dietary habits: “Are you vegetarian without eat meat, seafood for more than 5 years?” Those who answered “yes” were defined as vegetarians. The following question was asked regarding their daily consumption of milk, soymilk, calcium supplements, multiple vitamin, and B-complex vitamin intake: “Do you eat “…” every day?” Those who answered “yes” were defined as having a regular consumption habit. To reduce information bias regarding dietary habits, we confirmed them by double-checking with the patients and caregivers. Exercise habits of the study participants were defined using our questionnaire, which questioned past physical activity: “Did you practice sports or physical exercise sufficient to produce sweating or shortness of breath?” Those who answered “≥2.5 h per week” were defined as having exercise habits according to the previous study [7].

### 2.2. Cognitive Tests

A trained neuropsychologist administered neurobehavioral tests in the dementia integrated outpatient clinic. CDR scores [8] were used as a global assessment of cognitive and functional status. The CDR consists of six domains (memory, orientation, judgment and problem-solving, home and hobbies, community affairs, and personal care), each of which is scored as follows: “0” (no impairment), “0.5” (questionable impairment), “1” (mild impairment), “2” (moderate impairment), or “3” (severe impairment). The Mini-Mental State Examination (MMSE) [9] was also performed with all study participants.

### 2.3. Measurements of Vital Signs and Physical Condition

Blood pressure (BP) and heart rate status were recorded. Anthropometric measurements, including height and weight, were obtained and body mass index (weight [kg]/height [m^2^]) was estimated based on the measured height and weight. Each participant performed the Senior Fitness Test under supervision of a physical activity trainer. This test has been developed for the healthy elderly and people with dementia. It comprises six functional tests (the 30-s chair stand test, the 30-s arm curl test, the 2-min step test, the chair sit and reach test, the back scratch test, and the 8-foot up and go [8UG] test). In each testing session, seven testing stations were set up to each evaluate one item. Each testing item was conducted according to the specifications of the Senior Fitness Test Manual [10]. The 30-s chair stand test was defined as “number of full stands in 30 s with arms folded across chest”. The 30-s arm curl test was defined as “number of bicep curls in 30 s holding hand weight (women 5 lb; men 8 lb)”. The 2-min step test was defined as “number of full steps completed in 2 min, raising each knee to point midway between patella and iliac crest (score is number of times right knee reaches target)”. The chair sit and reach test was defined as “from sitting position at front of chair, with leg extended and hands reaching toward toes, number of inches (+ or −) from extended fingers to tip of toe”. The back scratch test was defined as “with one hand reaching over shoulder and one up middle of back, number of inches between extended middle fingers (+ or −)”, indicating a shoulder and upper body flexibility. The up and go [8UG] Test was defined as “number of seconds required to get up from seated position, walk 8 feet, turn, and return to seated position on chair”.

Handgrip strength was measured using the Jamar^®^ 5030J1 Hydraulic Hand Dynamometer (PATTERSON MEDICAL; Warrenville, IL, USA). Two consecutive measures of grip strength in each hand were recorded to the nearest kilogram (kg), with the participant in an upright position and the arm of the measured hand parallel to the body. Maximum grip strength was calculated by taking the average of the highest measurement from both hands.

### 2.4. Cognitive Decline Definition

According to a previous study [11], cognitive decline in cognitively normal individuals was defined as a decline in MMSE of at least 2 points after a 1-year interval of assessment.

### 2.5. Statistical Analysis

Statistical analyses were performed using IBM Statistical Product and Service Solutions (SPSS) software (version 25; SPSS, Inc., Armonk, NY, USA). Continuous variables were expressed as median with corresponding range, and were compared using the Mann–Whitney *U* test. Categorical variables were expressed in number and percentage, and were compared using Fisher’s exact test. Binary logistic regression analysis adjusted for possible correlations between factors and to determine any biomarkers associated with cognitive decline in participants with very mild dementia.

## 3. Results

### 3.1. Participant Characteristics

Demographic and clinical characteristics of all 132 study participants with very mild dementia are listed in Table 1. The participants were grouped into exercise (*n* = 99) and non-exercise (*n* = 33) groups, according to regular exercise habits (patients who had physical exercise ≥ 2 h per week were defined as having exercise habits); 54 (54%) females were in the exercise group and 23 (69%) females were in the non-exercise group. Compared to the exercise group, the non-exercise group had a higher admission rate (30% vs. 21%) and a higher proportion of participants with cognitive decline (33% vs. 28%), although these values were not statistically significant. The exercise group performed significantly better than the non-exercise group in the bilateral hand grip strength and Senior Fitness Test (except for the 8UG) (*p* < 0.05 for both comparisons). No significant between-group differences were observed for MMSE, BP, heart rate, or body weight measurements.

When we compared demographics and clinical characteristics between participants with or without rapid cognitive decline, the only variable found to be significantly associated with rapid cognitive decline was poorer performance in the right back scratch test, as shown in Table 2 (−14 [−39–37.5] cm vs. −17 [−57–7] cm; *p* = 0.038). In addition, the distribution of dietary habits, systemic diseases, and comorbidities were not significantly different between patients with/without cognitive decline (all *p* > 0.05, Table S1).

### 3.2. Association of Participant Characteristics and Cognitive Decline

A total of 39 participants exhibited a decline in MMSE of ≥2 points within 1 year of their initial assessment. After adjustment by age, a multivariate logistic regression analysis showed that right upper body flexibility (right back scratch test) was independently associated with cognitive decline in participants with very mild dementia (*p* = 0.022, Table 3).

## 4. Discussion

This exploration of factors that are possibly related to cognitive decline in patients with very mild dementia yielded the following novel key findings. First, participants with regular exercise habits performed better in all Senior Fitness Test categories and hand grip strength. Second, right back scratch (right shoulder/upper body flexibility) was independently correlated with cognitive decline, whereas age, sex, exercise habits, educational level, MMSE performance, body weight, grip strength, and other items in the Senior Fitness Test were not associated with rapid cognitive decline. Third, no associations were observed between dietary habits or systemic diseases with rapid cognitive decline in this study cohort.

Our study’s findings indicate that regular exercise improves physical strength and functional fitness. Similar outcomes in patients with arthritis of the hand demonstrate that exercises improve grip strength [12], while a 2010 Cochrane systemic review involving a total of 6700 older adults has suggested that progressive resistance training enhances gait speed, getting out of a chair, and muscle strength [13]. In another systemic review and meta-analysis of evidence from 43 randomized controlled trials (RCTs) involving 3988 participants with cognitive impairment and dementia, physical exercise training enhanced muscle strength, balance, mobility, and endurance, but weak evidence supported the use of exercise in improving flexibility [14]. According to these findings, we suggest that exercise may improve body strength, balance, and agility in patients with very mild dementia.

Although our data exhibit that regular exercise was not significantly associated with cognitive decline, a better understanding of the association between motor risk factors and cognitive performance in adults with cognitive impairment may assist with the early accurate detection of motor risk factors associated with cognitive decline in very mild dementia. Interestingly, a community-based cross-sectional study performed in Central China that included 2096 adults aged >65 years revealed that the relationship between functional fitness domains and cognitive impairment was independent of declining functional status [15], which is contrary to our study’s findings identifying better right back scratch (right shoulder/upper body flexibility) as the single variable to be associated with reduced cognitive decline. To our knowledge, our work is the first study to determine exercise-related senior fitness behaviors in patients with very mild dementia, as well as to identify potential factors associated with rapid cognitive decline. Few studies have investigated which type of fitness test may help to prevent cognitive decline. Recently, a study was reported by Kobayashi-Cuya et al. [16], who observed a significant association between cognitive decline and hand dexterity in older adults. Dexterity performance may relate to a better general fitness function and therefore reduced cognitive decline. For these findings, we recommend that a regular exercise may be a beneficial strategy to reduce cognitive decline because regular exercise is able to improve body strength, balance, and agility. Further studies may be conducted comparing cognitive decline speed for regular exercise and non-regular exercise groups.

A recent study has shown that the ipsilateral cortical representation of reaching arm movements differs strikingly between the left and right hemispheres, indicating that the left hemisphere shows better across-arm generalization [17]. Brain signals from the left but not from the right hemisphere correlate with inhibitory control, irrespective of which hand is employed [18]. Moreover, the right reaching arm movements have both a shorter movement time and stop signal reaction time than those with the left arm, which reflects a general function of the left hemisphere [19]. This can be explained by a greater specialization of the left hemisphere for controlling the magnitude and timing of muscular forces during movement execution [20]. Notably, patients with AD have exhibited predominant left rather than right hemisphere hypometabolism, which can be explained by greater susceptibility of the left hemisphere to degenerative or greater metabolic deficits resulting from left rather than right hemisphere impairment [21]. These findings led us to speculate that right shoulder/upper body flexibility-related cognitive decline exists in participants with very mild dementia due to a greater influence on specialization of the left hemisphere during movement execution or a greater degenerative and metabolic deficit in the left hemisphere.

Numerous studies have explored the factors associated with cognitive decline, including systemic diseases, biological factors, lifestyle, social economic status, and environment. A systemic review of evidence from 127 observational studies, 22 RCTs, and 16 systematic reviews that examined factors associated with risk for and possible prevention of cognitive decline in later life has suggested that cognitive training, physical exercise, vegetable intake, a Mediterranean-style diet, omega-3 fatty acids, and noncognitive and nonphysical leisure activities may lower the risk of cognitive decline in adults [22]. However, this finding was not observed in our population with very mild dementia, which failed to reveal any factors associated with rapid cognition decline. This implies that these risk factors may not affect cognitive decline in people with very mild dementia.

The findings of the current study need to be interpreted with caution. First, this was designed to be a prospective study in a single center with a relatively small sample size, and therefore we cannot establish a cause-and-effect relationship between hand dexterity and cognitive decline; longitudinal studies are needed to elucidate any such association. Second, more cognitive function tests should be used to provide further support to the association with hand dexterity. Third, due to the complexity of fine motor function, more shoulder/upper body flexibility tests are needed to better understand the association with executive function.

## 5. Conclusions

The right back scratch test (right shoulder/upper body flexibility) can serve as a crucial factor for predicting cognitive decline in patients with very mild dementia, as a protective effect upon cognitive decline. Although regular exercise was not significantly associated with cognitive decline, regular exercise may serve as an effective strategy for mitigating cognitive decline, as it has been shown to enhance better Senior Fitness Test performance and hand grip strength.

## Figures and Tables

**Table 1:** Baseline clinicodemographic profile of the 132 participants with very mild dementia included in the study.

	Median (Range)/*n* (%)
Age (years)	74 (53–88)
Sex (F/M)	77 (58.3%)/55 (41.7%)
Hospitalization after evaluation	31 (23.5%)
With regular exercise habit	99 (75%)
Education (years)	6 (0–22)
Mini-Mental State Examination	21 (10–30)
Systolic blood pressure (mmHg)	131 (94–181)
Diastolic blood pressure (mmHg)	71 (49–105)
Heart rate	78 (49–128)
Body height (cm)	158 (138–175)
Body weight (kg)	60.70 (36.00–97.60)
Body mass index (kg/m^2^)	24.20 (15.50–35.50)
Right grip strength (kg)	18 (0–40)
Left grip strength (kg)	18 (0–44)
Senior Fitness Test	
Chair stands (/30 s)	10 (0–50)
Right arm curls (/30 s)	13 (0–30)
Left arm curls (/30 s)	14 (0–36)
Step test (/2 min)	63 (0–144)
Right sit and reach (cm)	0 (−46–40.5)
Left sit and reach (cm)	0 (−42–37)
Right back scratch (cm)	−13 (−57–37.5)
Left back scratch (cm)	−23 (−80–40.5)
Up and go (s)	10 (0–90)

**Table 2:** Clinicodemographic data of the 132 participants with very mild dementia included in the study, categorized by rate of cognitive decline at baseline.

	With Rapid Cognitive Decline (*n* = 39)	Without Rapid Cognitive Decline (*n* = 93)
Age (years), median (range)	73 (53–86)	72.5 (61–88)
Sex (F/M), *n* (%)	19 (48.7%)/20 (51.3%)	58 (62.4%)/35 (37.6%)
With regular exercise habit, *n* (%)	28 (71.8%)	71 (76.3%)
Education (years), median (range)	6 (0–22)	6 (0–18)
Mini-Mental State Examination, median (range)	21 (11–30)	21 (10–29)
Systolic blood pressure (mmHg), median (range)	129 (94–173)	127 (100–181)
Diastolic blood pressure (mmHg), median (range)	73 (52–98)	70.5 (49–105)
Heart rate, median (range)	78 (59–128)	77 (49–109)
Body height (cm), median (range)	155.5 (146–174)	158 (138–175)
Body weight (kg), median (range)	60.25 (36.00–97.60)	60.20 (42.00–82.00)
Body mass index (kg/m^2^), median (range)	23.85 (15.50–34.30)	24.40 (18.80–35.50)
Right grip strength (kg), median (range)	18 (0–40)	21 (9–40)
Left grip strength (kg), median (range)	18 (4–32.5)	19 (0–44)
Senior Fitness Test		
Chair stands (/30 s), median (range)	10 (0–19)	11 (0–50)
Right arm curls (/30 s), median (range)	12 (0–30)	13.5 (0–27)
Left arm curls (/30 s), median (range)	14 (0–36)	14 (0–22)
Step test (/2 min), median (range)	62 (0–144)	65 (0–106)
Right sit and reach (cm), median (range)	0 (−40–20)	0 (−46–40.5)
Left sit and reach (cm), median (range)	0 (−38–20)	0 (−42–37)
Right back scratch (cm), median (range)	−14 (−39–37.5)	−17 (−57–7) *
Left back scratch (cm), median (range)	−23 (−4.9–40.5)	−25.5 (−80–8)
Up and go (s), median (range)	11 (0–58)	10 (0–90)

* *p* < 0.05 as compared to participants with rapid cognitive decline.

**Table 3:** Multivariate logistic regression analyses of factors associated with cognitive decline in patients with very mild dementia.

	Odds Ratio	95% Confidence Interval	*p*-Value
Age	1.043	0.963–1.129	0.297
Exercise	1.171	0.342–4.007	0.801
Vegetarian status	0.352	0.059–2.088	0.250
B-complex vitamin intake	0.957	0.290–3.152	0.942
Spinal operation history	2.434	0.663–1.071	0.180
Right grip strength (kg)	0.962	0.864–1.071	0.479
Left grip strength (kg)	0.986	0.875–1.112	0.818
Chair stands (/30 s)	1.013	0.851–1.205	0.886
Right arm curls (/30 s)	0.832	0.670–1.034	0.097
Left arm curls (/30 s)	1.202	0.935–1.544	0.151
Step test (/2 min)	1.008	0.983–1.034	0.534
Right sit and reach (cm)	0.980	0.854–1.125	0.776
Left sit and reach (cm)	1.015	0.879–1.171	0.842
Right back scratch (cm)	1.059	1.008–1.113	0.022 *
Left back scratch (cm)	0.978	0.939–1.020	0.300
Up and Go (s)	1.016	0.964–1.070	0.549

Binary logistic regression analysis considered exercise, vegetarian status, B-complex intake, and spinal operation history (category factor) as variable factors. * *p* < 0.05.

## Data Availability

The data used to support the findings of this study are included within the article and its Supplementary Materials.

## Supplementary Materials

The following supporting information can be downloaded at: https://www.mdpi.com/article/10.3390/jpm14111086/s1, Table S1: Systemic diseases recorded at 1 year of follow-up among the 132 study participants with very mild dementia.
