# Supplementary material for: A Simple Physical Examination Predicts Cognitive Decline in Very Mild Dementia
Source: J Pers Med. 2024 Nov 1;14(11):1086. doi: 10.3390/jpm14111086 (PMC11595368; doi:10.3390/jpm14111086)
Supplement: Supplementary file 1 [file jpm-14-01086-s001.zip › jpm-3219836-supplementary.pdf]

Table S1. Systemic diseases recorded at 1 year of follow-up among the 132 study participants with very mild dementia.

	With cognitive decline (n=39)	Without cognitive decline (n=93)
**Dietary habits**, n (%)		
Vegetarian	6 (15.3%)	14 (15.0%)
Mil	24 (61.5%)	33 (35.4%)
Soy mil	20 (51.2%)	28 (30.1%)
Calcium supplement	11 (28.2%)	20 (21.5%)
Multiple vitamin	6 (15.3%)	11 (11.8%)
B-complex	9 (23.0%)	13 (13.9%)
**Systemic diseases, comorbidities**, n (%)		
Diabetes mellitus	12 (30.7%)	21 (22.5%)
Hypertension	18 (46.1%)	35 (37.6%)
Hyperlipidemia	10 (25.6%)	11 (11.8%)
Gout	4 (10.2%)	5 (5.3%)
Knee operation history	2 (5.1%)	6 (6.4%)
Hip joint operation history	3 (7.6%)	2 (2.1%)
Spinal operation history	5 (12.8%)	6 (6.4%)
